# Production of Membrane Vesicles in *Listeria monocytogenes* Cultured with or without Sub-Inhibitory Concentrations of Antibiotics and Their Innate Immune Responses In Vitro

**DOI:** 10.3390/genes12030415

**Published:** 2021-03-13

**Authors:** Jung-Hwa Woo, Shukho Kim, Taewon Lee, Je-Chul Lee, Ji-Hyun Shin

**Affiliations:** 1 Department of Microbiology, School of Medicine, Kyungpook National University, Daegu 41944, Korea; dasomi999@naver.com (J.-H.W.); shukhokim@knu.ac.kr (S.K.); 2 Department of Applied Mathematics, College of Science and Technology, Korea University, Sejong 30019, Korea; taewon70@korea.ac.kr; 3 Institute of Science and Technology, College of Science and Technology, Korea University, Sejong 30019, Korea

**Keywords:** *Listeria monocytogenes*, membrane vesicles, innate immune response, antibiotics

## Abstract

Listeriosis is a food-borne illness caused by *Listeria monocytogenes*. Ampicillin (AMP) alone or in combination with gentamicin (GEN) is the first-line treatment option. Membrane vesicle (MV) production in *L. monocytogenes* under antibiotic stress conditions and pathologic roles of these MVs in hosts have not been reported yet. Thus, the aim of this study was to investigate the production of MVs in *L. monocytogenes* cultured with sub-minimum inhibitory concentrations (MICs) of AMP, GEN, or trimethoprim/sulfamethoxazole (SXT) and determine pathologic effects of these MVs in colon epithelial Caco-2 cells. *L. monocytogenes* cultured in tryptic soy broth with 1/2 MIC of AMP, GEN, or SXT produced 6.0, 2.9, or 1.5 times more MV particles, respectively, than bacteria cultured without antibiotics. MVs from *L. monocytogenes* cultured with AMP (MV_AMP_), GEN (MV_GEN_), or SXT (MV_SXT_) were more cytotoxic to Caco-2 cell than MVs obtained from cultivation without antibiotics (MV_TSB_). MV_AMP_ induced more expression of tumor necrosis factor (*TNF*)-*α* gene than MV_TSB_, MV_GEN_ and MV_SXT_, whereas MV_TSB_ induced more expression of interleukin (*IL*)-*1β* and *IL-8* genes than other MVs. Expression of pro-inflammatory cytokine genes by *L. monocytogenes* MVs was significantly inhibited by proteinase K treatment of MVs. In conclusion, antibiotic stress can trigger the biogenesis of MVs in *L. monocytogenes* and MVs produced by *L. monocytogenes* exposed to sub-MIC of AMP can induce strong pro-inflammatory responses by expressing *TNF-α* gene in host cells, which may contribute to the pathology of listeriosis.

## 1. Introduction

*L. monocytogenes* is a ubiquitous Gram-positive, non-spore-forming, facultative intracellular bacterium responsible for human listeriosis with high mortality in risk group, including pregnant women, the elderly, newborns, and immunocompromised individuals [1,2]. Effective antimicrobial therapy is essential to treat listeriosis. β-lactam antibiotics such as ampicillin (AMP), penicillin, and amoxicillin have been used for the treatment of listeriosis as the first-line option [3]. These antibiotics can penetrate into host cells where *L. monocytogenes* resides and bind to penicillin-binding protein 3 (involved in peptidoglycan biosynthesis) expressed by *L. monocytogenes* [4]. The antimicrobial dose is important when treating *L. monocytogenes* infections because penicillin exhibits bacteriostatic effects within cells only when cells are exposed to a high concentration. Depending on clinical conditions of infected patients, aminoglycoside, classically gentamicin (GEN), has been used in combination with ampicillin [5,6]. Trimethoprim/sulfamethoxazole (SXT) can also be used to treat listeriosis as a second-line option [7,8].

Bacterial extracellular vesicles (EVs) were first reported in *Escherichia coli* in the 1960s [9,10]. Since then, the production and biological roles of EVs have been reported in a variety of bacterial species. Secreted OMVs derived from Gram-negative bacteria are responsible for host cell cytotoxicity, the production of biofilms, transfer of antibiotic resistant genes, and modulation of innate immune responses in hosts [11,12]. Therefore, OMVs play an important role in the pathogenesis of Gram-negative bacterial pathogens. Production of membrane vesicles (MVs) and their biological roles in Gram-positive bacteria such as *Staphylococcus aureus* [13], *Bacillus* spp. [14], *Clostridium* spp. [15], and *L. monocytogenes* [16] have also been characterized. However, biological roles of Gram-positive bacteria-derived MVs involved in host–pathogen interaction as possible facilitators are less understood compared to those of Gram-negative bacteria-derived OMVs. In previous studies [16], we have demonstrated that *L. monocytogenes* can produce MVs during in vitro culture and that transcription factor σ^B^ plays a pivotal role in the biogenesis of MVs. Moreover, *L. monocytogenes* can produce more MVs under harsh conditions such as carbon starvation or salt stress conditions than under normal culture conditions [16,17]. Pathogenic bacteria, including *L. monocytogenes*, may encounter antibiotic stress conditions during infection. Gram-positive bacteria such as *Streptococcus pyogenes* and *S. aureus* can produce MVs actively under sub-inhibitory concentrations of β-lactam antibiotics [18,19]. In this circumstance, we postulate that sub-inhibitory concentrations of antibiotic stress may stimulate the production of MVs in *L. monocytogenes* and that these MVs may also contribute to pathogenesis with different aspects to host cells from MVs produced under conditions without antibiotics. Therefore, the aim of this study was to investigate the production of MVs in *L. monocytogenes* cultured with sub-minimum inhibitory concentrations (MICs) of AMP, GEN, or SXT and determine the ability of these MVs to induce cytotoxicity and innate immune responses in colon epithelial Caco-2 cells in vitro.

## 2. Materials and Methods

### 2.1. Bacterial Strain and Cell Culture

Wild-type *L. monocytogenes* 10403S strain (serotype 1/2a) was used in this study [16]. Bacteria were maintained on tryptic soy broth (TSB) (BD Science, Franklin Lakes, NJ, USA) at 37 °C. Human colon epithelial Caco-2 cells were obtained from the Korean Cell Line Bank (Seoul, Korea) and grown in Dulbecco’s modified Eagle medium (HyClone, Logan, UT, USA) supplemented with 10% fetal bovine serum (HyClone), 2 mM _L_-glutamine, 1000 U/mL penicillin G, and 50 μg/mL streptomycin at 37 °C in a humidified atmosphere with 5% CO_2_. Confluent cells were harvested and seeded into 24- or 96-well microplates for cell viability assays or 24-well plates for cytokine gene expression assays.

### 2.2. Antimicrobial Susceptibility Testing

MICs of AMP, GEN, and SXT (trimethoprim:sulfamethoxazole, 1:5) were determined by microdilution methods according to the Clinical Laboratory Standards Institute (CLSI) [20]. *S. aureus* ATCC 29213 was used as a quality control strain.

### 2.3. Isolation of MVs

MVs produced by *L. monocytogenes* were isolated from bacterial culture supernatants as previously described [16]. Briefly, *L. monocytogenes* was cultured in 500 mL of TSB at 37 °C with shaking (180 rpm). Bacteria were cultured in 500 mL of TSB supplemented with 1/2 MIC of AMP (0.25 µg/mL), GEN (0.25 µg/mL), or SXT (0.25/1.25 µg/mL) at 37 °C with shaking to isolate MVs from *L. monocytogenes* under antibiotic stress conditions. Bacteria were cultured to late exponential phase at OD_600_ of 1.4. Cultured bacterial cells were then centrifuged at 6000× *g* for 15 min at 4 °C. Culture supernatants were filtered using a QuixStand Benchtop System (GE Healthcare, Amersham, UK) with a 0.2 µm hollow fiber membrane (GE Healthcare) and concentrated using a QuixStand Benchtop System with a 500 kDa hollow fiber membrane (GE Healthcare). MV samples were collected by ultracentrifugation at 150,000× *g* for 3 h at 4 °C and washed in phosphate-buffered saline (PBS) followed by another ultracentrifugation. MV pellets were resuspended in PBS. Purified MVs were streaked onto blood agar plates to check for sterility and then stored at −80 °C until use.

### 2.4. Nano Particle Tracking Analysis (NTA)

MV size and concentration were measured using a NanoSight NS500 instrument with a 488 nm laser module and sCMOS camera module (Malvern Instruments, Worcestershire, UK). The small volume of MV samples obtained from ultracentrifugation was serially diluted in MilliQ water and then we preliminarily checked the numbers of MV particles in the diluted samples. After calculation of dilution factor to obtain approximately 8~9 × 10^8^ particles/mL, MV samples were diluted in MilliQ water to such concentration and then NTA was performed to determine MV size and number of particles. NTA measurement yielded 50~100 particles per frame. Samples were loaded into the sample chamber. Videos were then recorded for 30 s three times. Captured data were analyzed using NTA 3.1 software build 3.1.46. All measurements were performed in triplicate at room temperature.

### 2.5. Cell Viability Assay

The viability of Caco-2 cells treated with *L. monocytogenes* MVs was measured using 3-[4,5-dimethylthiazol-2-yl]-2,5 diphenyltetrazolium bromide (MTT) assay (Abcam, Cambridge, UK). Live and dead bacteria were prepared to measure their cytotoxicities to Caco-2 cells. *L. monocytogenes* was cultured in TSB (OD_600_ of 1) to prepare live bacteria. Dead bacteria were prepared by fixing *L. monocytogenes* cells with 10% formalin for 20 h and then washed with PBS five times by centrifugation at 13,000 rpm for 10 min. Bacterial viability was checked on blood agar plates. Briefly, Caco-2 cells were seeded into 24- or 96-well microplates and then treated with different concentrations (1~20 μg/mL) of MVs for 24 h. Absorbance was measured at 570 nm after treatment with MTT reagent for 2 h. The cell viability assay was performed in three independent experiments.

### 2.6. Expression of Pro-Inflammatory Cytokine Genes Determined by Quantitative Real-Time Polymerase Chain Reaction (qPCR)

Expression levels of genes encoding glyceraldehyde 3-phosphate dehydrogenase (GAPDH), interleukin (*IL*)-*1β*, *IL-8*, and tumor necrosis factor (*TNF*)-*α* were assessed by qPCR as previously described [21]. Caco-2 cells (2.0 × 10^5^ cells/mL) were treated with 1, 2, or 5 μg/mL of *L. monocytogenes* MVs for 4 h. Total RNAs were extracted using an RNeasy Mini Kit (Qiagen, Valencia, CA, USA). cDNAs were then synthesized from 1 μg of total RNAs using oligo dT primers and M-MLV reverse transcriptase (Fermentas) in a total reaction volume of 40 μL. Gene expression was quantified using TOPreal^TM^ qPCR 2X PreMIX (SYBR Green with high ROX) (Enzynomics) on an ABIPRISM 7500 Real-Time System (Applied Biosystems, Foster City, CA, USA). Fold changes in gene expression were calculated using the comparative Ct method. Expression levels of genes were normalized to GAPDH expression levels. Each experiment was performed in triplicate.

### 2.7. Treatment of L. monocytogenes MVs with Proteinase K and Lysozyme

Purified *L. monocytogenes* MVs were treated with 0.1 mg/mL proteinase K (Biofact, Daejeon, Korea) at 50 °C for 3 h to degrade MV proteins or 1 mg/mL lysozyme (Sigma-Aldrich, St. Louis, MO, USA) at 37 °C for 30 min to degrade MV peptidoglycans.

### 2.8. Statistical Analysis

Data were analyzed using R 3.3.4 (https://www.r-project.org/, accessed on 15 November 2020). Expression levels of genes were analyzed using a one-way analysis of variance (ANOVA) with Dunnett’s post hoc analysis. Student’s *t*-test was used to determine statistical significance of cell viability and cytokine gene expression between intact MVs and proteinase K- or lysozyme-treated MVs. Differences were considered statistically significant at *p* < 0.05.

## 3. Results

### 3.1. MV Production in L. monocytogenes Cultured with Sub-Inhibitory Concentrations of Antibiotics

**Figure 1:**
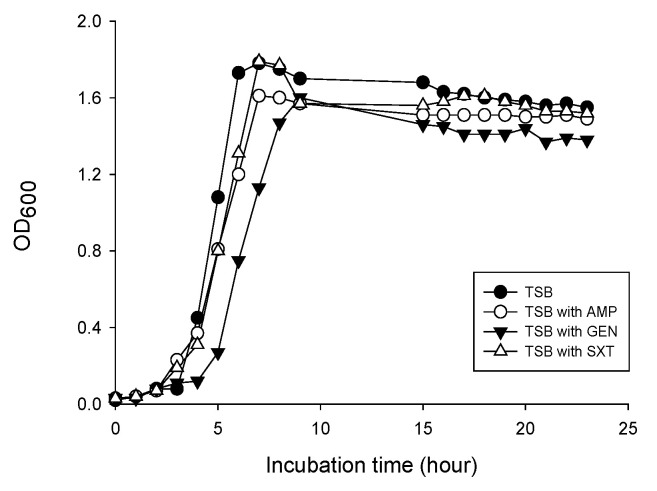
Growth of *L. monocytogenes* under antibiotic stress conditions. Bacteria were cultured in tryptic soy broth (TSB) without antibiotics, TSB with 0.25 μg/mL ampicillin (AMP), TSB with 0.25 μg/mL gentamicin (GEN), or TSB with 0.25/1.25 μg/mL trimethoprim/sulfamethoxazole (SXT) at 37 °C with shaking. Bacterial growth was measured at OD_600_ at indicated time points. Data are representative of three independent experiments with similar results.

To determine effects of sub-inhibitory concentrations of antibiotics on MV production, *L. monocytogenes* was cultured in TSB without antibiotics or TSB supplemented with 1/2 MIC of AMP, GEN, or SXT with shaking to reach late exponential phase at OD_600_ of 1.4 (Figure 1).

**Figure 2:**
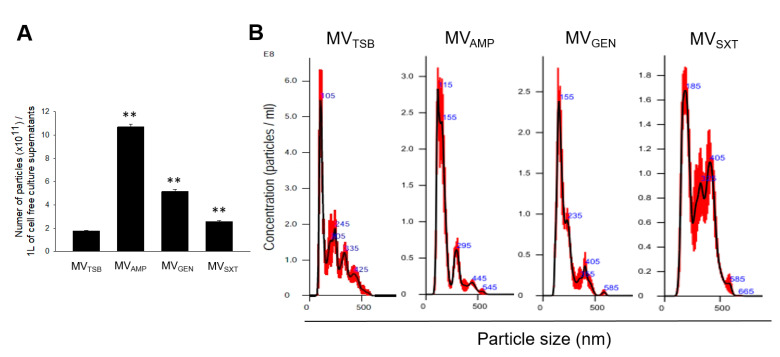
Production of membrane vesicles (MVs) in *L. monocytogenes* cultured under antibiotic stress conditions. Bacteria were grown to late exponential phase (OD_600_ of 1.4). MVs were then isolated from bacterial culture supernatants. Nano particle tracking analysis (NTA) was performed to determine the number and size of MVs. (**A**) Particle numbers of MV_TSB_, MV_AMP_, MV_GEN_, and MV_SXT_ in 1 L of cell free culture supernatants. Data are presented as mean ± SD of three independent experiments. **, *p* < 0.01 compared to MV_TSB_. (**B**) Size distribution of MV_TSB_, MV_AMP_, MV_GEN_, and MV_SXT_ based on NTA. Data are representative of three independent experiments with similar results.

MVs were isolated from bacterial culture in TSB without antibiotics (MV_TSB_) or in TSB with AMP (MV_AMP_), with GEN (MV_GEN_), or with SXT (MV_SXT_). MV samples of MV_TSB_, MV_AMP_, MV_GEN_, and MV_SXT_ obtained from 1 L of cell free culture supernatants contained 1.78 × 10^11^, 10.7 × 10^11^, 5.15 × 10^11^, and 2.59 × 10^11^ particles, respectively, based on NTA (Figure 2A). Mean sizes of MV_TSB_, MV_AMP_, MV_GEN_, and MV_SXT_ were 106.3 ± 3.7 nm, 132.6 ± 12.4 nm, 156.9 ± 6.9 nm, and 194.4 ± 14.8 nm, respectively (Figure 2B). These results suggest that sub-inhibitory concentrations of antibiotics such as AMP, GEN, and SXT used to treat listeriosis can affect the biogenesis of MVs in *L. monocytogenes.*

### 3.2. Host Cell Cytotoxicity Induced by MVs from L. monocytogenes Cultured with Sub-Inhibitory Concentrations of Antibiotics

**Figure 3:**
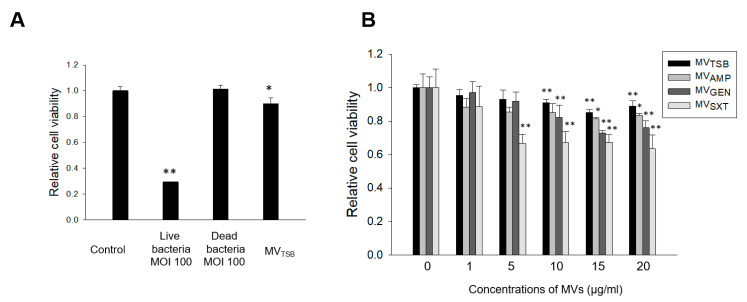
Cytotoxicities of bacteria and MVs from *L. monocytogenes* cultured with or without antibiotics to Caco-2 cells. (**A**) *L. monocytogenes* was cultured in TSB to reach 1.0 at OD_600_. Caco-2 cells were then treated with live bacteria at multiplicity of infection (MOI) 100, dead bacteria at MOI 100, and MV_TSB_ (10 μg/mL) for 24 h. Dead bacteria were prepared by fixation with 10% formalin for 20 h. (**B**) Caco-2 cells were treated with various concentration of MV_AMP_, MV_GEN_, or MV_SXT_ for 24 h. Cell viability was determined using an MTT assay. Data are presented as mean ± SD of three independent experiments. *, *p* < 0.05; **, *p* < 0.01 compared to untreated control cells.

To determine whether live *L. monocytogenes*, dead *L. monocytogenes*, and bacteria-derived MVs could induce cytotoxicity to human colon epithelial cells, Caco-2 cells were treated with live bacteria at multiplicity of infection (MOI) 100, dead bacteria at MOI 100, or 10 μg/mL of MV_TSB_ for 24 h. Cell viability was then analyzed using the MTT assay. Results revealed that live bacteria and 10 μg/mL of MV_TSB_, but not dead bacteria, induced cytotoxicity to Caco-2 cells (Figure 3A). Next, to determine whether *L. monocytogenes* MVs could induce cytotoxicity in a dose-dependent manner, Caco-2 cells were treated with various concentrations (1~20 μg/mL) of different MVs for 24 h. No cytotoxicity was observed for cells treated with 1/2 MICs of AMP, GEN, and SXT for 24 h (Figure S1). Cytotoxicity was induced in Caco-2 cells treated with ≥5 µg/mL of MV_SXT_, ≥10 µg/mL of MV_GEN_ and MV_TSB_, and ≥15 µg/mL of MV_AMP_ when the cytotoxicity was compared to untreated (no antibiotics) control cells (Figure 3B). MVs derived from *L. monocytogenes* cultured under three antibiotic stress conditions were more cytotoxic at ≥ 10 µg/mL than MV_TSB_ at the same concentration. Among different MVs, MV_SXT_ exhibited the highest cytotoxicity. These results suggest that *L. monocytogenes* exposed to sub-inhibitory concentrations of SXT can produce more cytotoxic MVs than bacteria under exposure to sub-inhibitory concentrations of AMP or GEN or no antibiotics.

### 3.3. Difference in Ability to Induce an Innate Immune Response by MVs from L. monocytogenes Cultured with Sub-Inhibitory Concentrations of Antibiotics

**Figure 4:**
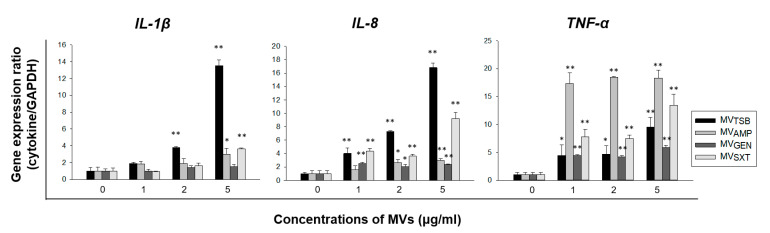
Expression levels of pro-inflammatory cytokine and chemokine genes in Caco-2 cells treated with *L. monocytogenes* MVs produced with or without antibiotics. Caco-2 cells were treated with various concentrations of *L. monocytogenes* MVs for 4 h. Gene expression was then assessed via qPCR. Data are presented as mean ± SD of three independent experiments. *, *p* < 0.05; **, *p* < 0.01 compared to untreated control cells.

To determine whether MVs from *L. monocytogenes* cultured under antibiotic stress conditions could trigger an innate immune response in vitro, Caco-2 cells were treated with 1, 2, or 5 μg/mL of MV_TSB_, MV_AMP_, MV_GEN_, or MV_SXT_ for 4 h. Expression levels of pro-inflammatory cytokine genes (such as *IL-1β* and *TNF-α*) and chemokine gene such as *IL-8* were analyzed using qPCR. As shown in Figure 4, MV_TSB_ significantly stimulated the expression of *IL-1β* and *IL-8* genes compared with other MVs, whereas the expression level of *TNF-α* gene was significantly higher in cells treated with MV_AMP_ than in cells treated with MV_TSB_, MV_GEN_, or MV_SXT_. MV_SXT_ induced more expression of *TNF-α* gene than MV_TSB_. These results suggest that sub-inhibitory concentrations of antibiotics can alter MV-induced pro-inflammatory responses in Caco-2 cells. Furthermore, sub-inhibitory concentrations of AMP affected the ability of *L. monocytogenes* MVs to induce pro-inflammatory response by altering the expression level of *TNF-α* in Caco-2 cells.

### 3.4. MV Components Responsible for Cytotoxicity and Innate Immune Response in Host Cells

**Figure 5:**
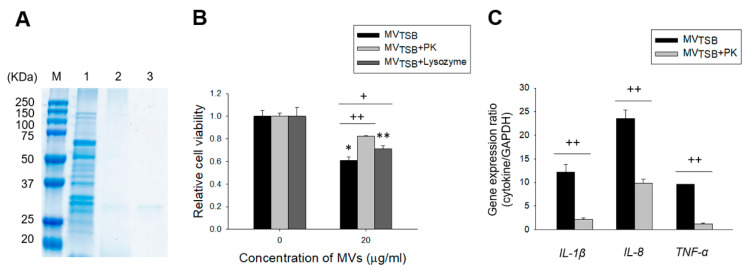
Host cell responses to proteinase K-treated *L. monocytogenes* MVs. (**A**) SDS-PAGE analysis of MV proteins. *L. monocytogenes* MVs were treated with 0.1 mg/mL proteinase K for 3 h at 50 °C for the degradation of MV proteins. M, molecular weight marker; 1, intact MV_TSB_; 2, proteinase K-treated MV_TSB_; 3, proteinase K. (**B**) Caco-2 cells were incubated with intact (MV_TSB_), proteinase K-treated MVs (MV_TSB_ + PK), or lysozyme-treated MVs (MV_TSB_ + Lysozyme) for 24 h. Cell viability was then determined using an MTT assay. Data are presented as mean ± SD of three independent experiments. *, *p* < 0.05; **, *p* < 0.01 compared to untreated control cells. +, *p* < 0.05; ++, *p* < 0.01 compared to 20 µg/mL of MV_TSB_. (**C**) Caco-2 Cells were incubated with intact or proteinase K-treated MV_TSB_ for 4 h. Gene expression was then assessed using qPCR. Data are presented as mean ± SD of three independent experiments. ++, *p* < 0.01 compared to intact MV_TSB_.

To determine which *L. monocytogenes* MV components (proteins or peptidoglycans) were responsible for the cytotoxicity and innate immune response in host cells, MV_TSB_ were treated with proteinase K or lysozyme. As shown in Figure 5A, proteins in MV_TSB_ were degraded by proteinase K treatment. After Caco-2 cells were incubated with intact, proteinase K-treated, or lysozyme-treated MV_TSB_ for 24 h, cytotoxicity to Caco-2 cells was significantly decreased for MV_TSB_ incubated with proteinase K or lysozyme compared to intact MV_TSB_ (Figure 5B). These results suggest that both proteins and peptidoglycans in MVs are responsible for the cytotoxicity of MVs to host cells. Next, Caco-2 cells were incubated with intact or proteinase K-treated MV_TSB_ for 4 h. Expression levels of *IL-1β*, *IL-8*, and *TNF-α* genes were then assessed using qPCR. Expression levels of all tested cytokine genes were significantly decreased in cells incubated with proteinase K-treated MV_TSB_ as compared to those in cells incubated with intact MV_TSB_ (Figure 5C). These results suggest that proteins in MVs are partly responsible for the pro-inflammatory responses in Caco-2 cells.

## 4. Discussion

The present study demonstrated that *L. monocytogenes* exposed to sub-inhibitory concentrations of antibiotics used to treat listeriosis produced more MVs than bacteria cultured under no antibiotics in vitro. Furthermore, MVs obtained from bacterial culture under different antibiotics induced different host cell responses regarding cytotoxicity and pro-inflammatory responses. These results suggest that sub-inhibitory concentrations of antibiotics may alter MV-mediated host pathology involved in *L. monocytogenes* infection.

In previous studies, we have shown that *L. monocytogenes* can produce MVs during in vitro culture and that this microorganism can actively utilize MVs as a secretion system to interact with host cells or as a cargo of virulence factors [16,17,21]. The size, shape, and amount of *L. monocytogenes* MVs varied depending on transcription factor σ^B^, bacterial growth stage, and stress type [16,17]. Sigma B is directly responsible for the production of *L. monocytogenes* MVs [16]. Wild-type strain produced nine times more MVs than Δ*sigB* mutant strain cultured under carbon starvation stress conditions, whereas salt stress condition did not affect MV production [16,17]. In the present study, sub-inhibitory concentration of antibiotics such as AMP, GEN, or SXT stimulated the production of *L. monocytogenes* MVs. Especially, bacteria cultured in the presence of AMP produced six times more MV particles than bacteria cultured in the presence of GEN or SXT or no antibiotics (Figure 2A). AMP can inhibit bacterial cell wall synthesis [4]. Biagini et al. [18] have shown that sub-inhibitory concentration of penicillin can stimulate MV production in *S. pyogenes* by weakening cell walls. Cell wall inhibitors such as β-lactams exhibit more profound effects on MV production in *L. monocytogenes* than protein synthesis inhibitor GEN or folate pathway inhibitor SXT. In addition, MV size in *L. monocytogenes* was different between antibiotic and no antibiotic conditions. MV_TSB_ sizes were measured to have an average of 106 nm in diameter, whereas sizes of MVs produced by *L. monocytogenes* under antibiotic stress conditions ranged from 132 to 194 nm in diameter (Figure 2B). This suggests that MV sizes produced under antibiotic stress conditions are much bigger than MVs produced under no antibiotics. Interestingly, MV_SXT_ exhibited the largest size among different MVs. In Gram-negative bacterium *Burkholderia cepacia*, MVs obtained from bacterial culture under sub-inhibitory concentrations of SXT also showed the biggest size compared to MVs from bacterial culture under sub-inhibitory concentrations of meropenem or ceftazidime [22]. These results suggest that sub-inhibitory concentrations of folate pathway inhibitors may produce bigger size of EVs in both Gram-positive and Gram-negative bacteria than other classes of antibiotics. Our results suggest that *L. monocytogenes* can regulate the biogenesis of MVs in different ways when bacteria are exposed to different antibiotics.

We have previously shown that MVs isolated from *L. monocytogenes* cultured with or without salt stress to early exponential phase (OD_600_ of 0.5) are not cytotoxic to Caco-2 cells, although they can increase cell viability when cells are treated with MVs ≤ 30 μg/mL [21]. However, in the present study, MVs from *L. monocytogenes* cultured with or without antibiotics to late exponential phase (OD_600_ of 1.4) did induce cytotoxicity to Caco-2 host cells in a dose-dependent manner (Figure 3B). Furthermore, the ability of MVs to induce cytotoxicity was different between MVs obtained from different antibiotic stress conditions. MVs produced under antibiotic stress conditions (MV_AMP_, MV_GEN_, and MV_SXT_) were more cytotoxic than MVs produced under no antibiotic conditions. MV_SXT_ showed the highest cytotoxicity among four *L. monocytogenes* MVs. We have previously shown that salt stress can regulate global protein profile of *L. monocytogenes* MVs [17]. These results suggest that bacterial culture conditions or bacterial growth stages may regulate molecular components associated with cytotoxicity of *L. monocytogenes* MVs to host cells. As shown in Figure 5B, cytotoxicities of both proteinase K- and lysozyme treated-*L. monocytogenes* MVs to host cells were significantly reduced compared to those of intact MVs. Twenty μg/mL of MV_TBS_ significantly induced cell cytotoxicity. However, relative cell viability was lower than that shown in Figure 3B (0.61 ± 0.03 vs. 0.89 ± 0.03). The size of the microplate (96 vs. 24-well microplate) used to measure cytotoxicity might has an influence on the difference of viabilities. These results indicate that proteins and peptidoglycans are directly responsible for the cytotoxicity of *L. monocytogenes* MVs. Although specific cytotoxic factors in *L. monocytogenes* MVs were not identified in this study, *L. monocytogenes* exposed to sub-inhibitory concentrations of antibiotics produced more cytotoxic MVs than bacteria under no antibiotic conditions.

Interaction of *L. monocytogenes* with intestinal epithelial cells can stimulate the production of pro-inflammatory cytokines such as *TNF-α* and *IL-6* and chemokines such as *IL-8* and monocyte chemoattractant protein (*MCP*)-1 to initiate mucosal inflammatory response [23]. In our previous study [21], we have shown that *L. monocytogenes* MVs (10 μg/mL) can stimulate the expression of *IL-1β*, *IL-6*, *TNF-α*, *IL-8*, *MCP-1*, and *MIP-1α* genes in Caco-2 cells. Interestingly, expression levels of *IL-1β*, *IL-6*, and *TNF-α* genes in cells treated with MVs produced under no salt stress condition were significantly higher than those in cells treated with MVs produced under salt stress condition. However, expression levels of chemokine genes including *IL-8*, *MCP-1*, and *MIP-1α* were higher in cells treated with MVs produced under salt stress than in cells treated with MVs produced under no salt stress. In the present study, we determined whether *L. monocytogenes* MVs produced under antibiotic stress conditions stimulated the expression of *IL-1β*, *TNF-α*, and *IL-8* genes in Caco-2 cells. In agreement with the previous study, MV_TSB_ stimulated the expression *IL-1β*, *TNF-α*, and *IL-8* genes, although expression levels of these genes in Caco-2 cells treated with MVs produced with antibiotics were significantly different from those in Caco-2 cells treated with MVs produced without antibiotics. Expression levels of *IL-1β* and *IL-8* genes were the highest level in cells treated with MV_TSB_, whereas the expression level of *TNF-α* gene was the highest in cells treated with MV_AMP_ (Figure 4). These results suggest that antibiotic stress may alter the pathogenesis of *L. monocytogenes* by regulating pro-inflammatory responses in epithelial cells responding to secreted MVs. MVs produced by Gram-positive bacteria contain many pathogen-associated molecular patterns (PAMPs) such as peptidoglycans, proteins, and nucleic acid [13,24,25]. These PAMP molecules can be recognized by pattern recognition receptors expressed in host cells and activate the innate immune response through the induction of a variety of pro-inflammatory cytokines [26]. As shown in Figure 5C, MV_TSB_ treated with proteinase K significantly inhibited the expression of *IL-1β*, *TNF-α*, and *IL-8* genes as compared to intact MVs. Treatment of MVs derived from *Enterococcus faecium* with proteinase K also significantly reduced the expression of pro-inflammatory cytokine and chemokine genes in Caco-2 cells as compared to treatment with intact MVs [27]. These findings suggest that proteins in the surface or lumens of *L. monocytogenes* MVs are largely responsible for pro-inflammatory responses in host cells. However, other components in MVs involved in pro-inflammatory responses need to be identified because expression levels of *IL-1β*, *IL-8*, and *TNF-α* genes are not completely inhibited by proteinase K-treated MVs. Our results support that gastrointestinal stresses such as salt or antibiotics may contribute to the diversity of PAMPs or cargo molecules in *L. monocytogenes* MVs involved in pro-inflammatory responses in host cells.

To treat listeriosis, antimicrobial is essential. However, resistance of *L. monocytogenes* to commonly used antibiotics has emerged [5,28,29]. The first antibiotic-resistance strain of *L. monocytogenes* was reported in 1988 [30]. Since then, many resistant strains have been isolated from food, environment, and clinical cases. A recent study has reported that most of isolates of multidrug-resistant *L. monocytogenes* strains isolated from beef cattle and are resistant to AMP, penicillin, and erythromycin [31]. The present study analyzed the pathology of host cells induced by MVs secreted from *L. monocytogenes* cultured under different antibiotic conditions. Sub-inhibitory concentrations of AMP, GEN, and SXT increased the production of MVs in *L. monocytogenes*. Especially, MV_SXT_ exhibited a strong cytotoxic activity towards intestinal epithelial cells, whereas MV_AMP_ induced strong pro-inflammatory responses via the expression of *TNF-α* gene. Our findings may help us understand the biogenesis of MVs and their contribution to pathogenesis of *L. monocytogenes* under antibiotic stress conditions that may encounter during an in vivo infection. The challenge of future studies is to identify cargo molecules associated with host cell cytotoxicity and pro-inflammatory responses in *L. monocytogenes* MVs.

## Supplementary Materials

The following are available online at https://www.mdpi.com/2073-4425/12/3/415/s1, Figure S1: Viabilities of Caco-2 cells treated with 1/2 MICs of antibiotics.
