# Supplementary material for: Production of Membrane Vesicles in Listeria monocytogenes Cultured with or without Sub-Inhibitory Concentrations of Antibiotics and Their Innate Immune Responses In Vitro
Source: Genes (Basel). 2021 Mar 13;12(3):415. doi: 10.3390/genes12030415 (PMC7998634; doi:10.3390/genes12030415)
Supplement: Supplementary file 1 [file genes-12-00415-s001.pdf]

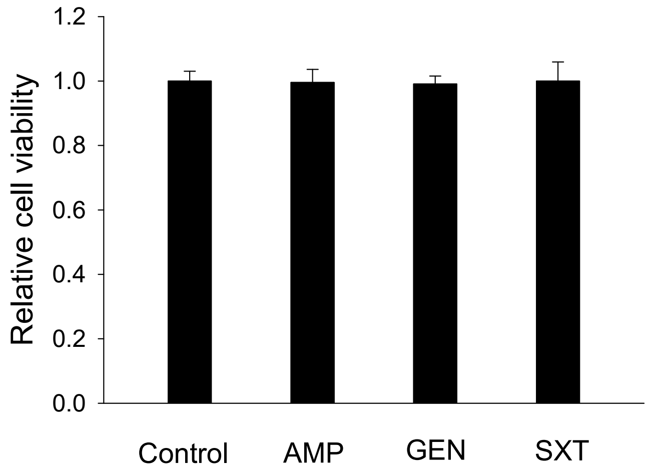

Figure S1. Viabilities of Caco-2 cells treated with 1/2 MICs of antibiotics. Caco-2 cells were treated with 0.25 μg/ml ampicillin (AMP), 0.25 μg/ml gentamicin (GEN), or 0.25/1.25 μg/ml trimethoprim/sulfamethoxazole (SXT) for 24 h. Cell viability was determined using an MTT assay. Antibiotics were not cytotoxic to Caco-2 cells.
